# The synthesis and highly effective antibacterial properties of Cu-3, 5-dimethy l-1, 2, 4-triazole metal organic frameworks

**DOI:** 10.3389/fchem.2023.1124303

**Published:** 2023-02-15

**Authors:** Xiaolin Xu, Mengna Ding, Kaiquan Liu, Fujian Lv, Yingchun Miao, Yanmi Liu, Ying Gong, Yuning Huo, Hexing Li

**Affiliations:** ^1^ Faculty of Chemical and Environmental Science, Key Laboratory of Environment Chemistry, Qujing Normal University, Qujing, Yunnan, China; ^2^ The Education Ministry Key Lab of Resource Chemistry, Joint International Research Laboratory of Resource Chemistry, Ministry of Education, Shanghai Key Laboratory of Rare Earth Functional Materials, Shanghai Frontiers Science Research Base of Biomimetic Catalysis, and College of Chemistry and Materials Science, Shanghai Frontiers Science Center of Biomimetic Catalysis, Shanghai Normal University, Shanghai, China

**Keywords:** Cu-3,5-dimethyl-1,2,4-triazole MOFs, electrostatic interaction, sterilization, inhibition-zone diameter, *Staphylococcus Aureus*

## Abstract

The influence of metal ions, the state of metal salt, and ligands on the sterilization ability of (Metalorganic frameworks) MOFs to effectively achieve sterilization has been investigated in this study. Initially, the MOFs were synthesized by elements of Zn, Ag, and Cd for the same periodic and main group of Cu. This illustrated that the atomic structure of Cu was more beneficial for coordinating with ligands. To further induce the maximum amount of Cu^2+^ ions in the Cu-MOFs to achieve the highest sterilization, various Cu-MOFs synthesized by the different valences of Cu, various states of copper salts, and organic ligands were performed, respectively. The results demonstrated that Cu-MOFs synthesized by 3, 5-dimethyl-1, 2, 4-triazole and tetrakis (acetonitrile) copper(I) tetrafluoroborate presented the largest inhibition-zone diameter of 40.17 mm towards *Staphylococcus Aureus* (*S. aureus*) under dark conditions. The proposed mechanism of Cu (Ⅱ) in MOFs could significantly cause multiple toxic effects, such as the generation of reactive oxygen species, and lipid peroxidation in *S. aureus* cells, when the bacteria was anchored by the Cu-MOFs *via* electrostatic interaction. Finally, the broad antimicrobial properties of Cu-MOFs against *Escherichia coli* (*E. coli*), *Acinetobacter baumannii* (*A. baumannii*), and *S. aureus* were demonstrated. In conclusion, the Cu-3, 5-dimethyl-1, 2, 4-triazole MOFs appeared to be potential antibacterial catalysts in the antimicrobial field.

## 1 Introduction

Diseases induced by bacteria have become a serious threat to human health and arisen great attention (Ayoub Moubareck and Hammoudi Halat, 2020). At present, many developed antibacterial methods mainly focus on inhibiting the survival of bacteria by randomly destroying their membrane. These include ultraviolet sterilization (Wegener et al., 2017), antibacterial ozone (Dodd et al., 2009), antibacterial chlorides (Zhang X. et al., 2022), and antibiotics (Wang et al., 2020). However, with widely-accepted disadvantages of drug resistance, high energy cost, and secondary pollution to the environment, these methods have limited applications (Ding et al., 2020). Thus, it is significant to develop a safe and efficient novel antibacterial agent to meet the demands of human health.

Metal-organic frameworks (MOFs) are promising developed inorganic-organic hybrid porous materials which have been regarded as a potential antibacterial material to be utilized in biological (Pettinari et al., 2021), environmental (Wang et al., 2022), and food antimicrobial fields (Zhang M. et al., 2022). Especially, MOFs have attracted tremendous attention in antibacterial fields in the last few decades owing to their porosity, sustained release capability, and structural flexibility. Many antibacterial composite MOF materials have been explored by combining a variety of chemicals and antibacterial materials, such as nanoparticles (Duan et al., 2017), antibiotics (Li et al., 2022), phytochemicals (Wu et al., 2019), and polymers (Gowriboy et al., 2022). These include Ag-CuTCPP (Ximing et al., 2017), Ag@ZIF-8 (Barjasteh et al., 2022), the encapsulation of ciprofloxacin into ZIF-8, RFP&o-NBA@ZIF-8 (Song et al., 2018), and Ag-MOFs-polyetherimide (PEI) (Li et al., 2015). Metal ions and ligands for bacterial inactivation have been regarded as the main significant factors to affect the sterilization ability of MOFs. However, there are few systematic studies on the influence of metal ions (transition metal elements of the IB and IIB of sub-family), the state of metal salt (organic or inorganic salt), and ligands (imidazole, pyridine, and carboxylic acid) on the sterilization ability of MOFs.

Additionally, the antibacterial mechanism of MOFs has been proposed in recent years. MOF materials have excellent physical-chemical properties that could avoid the disadvantage of drug resistance of antibiotics (Li et al., 2022) and generate effective antibacterial capability. They mainly rely on physical contact between the MOFs and bacteria *via* van der Waals forces (Vecitis et al., 2010), electrostatic interaction (Miao et al., 2019), and hydrophobic interactions (Pang et al., 2017). As a result, the contact leads to bacterial death by inducing cell membrane cracks and intracellular substance inactivation (Vecitis et al., 2010). Another physical contact mechanism of the antimicrobial route of MOFs illustrated is hybrid nanocomposites, such as GO/Co-MOFs, which effectively destroy bacterial membranes with sharp edges, resulting in cell inactivation by leakage of intracellular substances, such as K^+^, DNA, functional proteins, and enzymes (Pang et al., 2017). Moreover, MOFs can be utilized as a metal storage container for the slow release of Ag, Co, Cu, and Ni ions, providing a durable antibacterial ability (Liu et al., 2021). The released metal ions could permeate into the cell membrane slowly and destroy cellular components (Liu et al., 2021). Different metal ions exhibit various inactivation mechanisms. For example, the noble Ag^+^ ions could result in bacterial inactivation by splitting the membrane and destroying the nucleic acid, enzymes, and blocking-up electron transport (Miller et al., 2015). Cu^2+^ ions dispersed in an aqueous solution could possess the capability to generate ROS to disrupt the synthesis of DNA (Hajipour et al., 2012) and amino acids. Released Co^2+^ ions are able to produce pro-oxidative stress and reactive oxygen species ROS by interacting with the PO_4_^3-^ of the phospholipids in the membrane of bacteria (Zhuang et al., 2012). Ligand molecules with antimicrobial properties can be located inside the spatial structure of MOFs to combine uniformly distributed metal ions to form a synergistic effect of antibacterial activity (Liu et al., 2021). According to investigations, it has been discovered that various antibacterial mechanisms are proposed for different MOFs. These mechanisms are not universal and do not have a fixed probability for all the MOFs. Additionally, the Cu-3, 5-dimethyl-1,2, 4-triazole metal organic framework was proved to possess a highly effective inhibition effect during previous systematic investigation. Therefore, it is essential and necessary to explore its antibacterial mechanism.

Herein, the various Cu-MOFs were developed by different organic copper salt and ligands. Zn-MOFs, Cd-MOFs, and Ag-MOFs were successfully synthesized at room temperature and were antimicrobial for *S. aureus* during both the dark and visible light systems. It was discovered that the Cu-3, 5-dimethyl-1, 2, 4-triazole metal-organic framework presented the most significant inhibition effect. Thus, *E. coli* and *A. baumannii* were introduced to study the framework’s broad antimicrobial properties. Finally, the antibacterial mechanism of Cu-3, 5-dimethyl-1, 2, 4-triazole metal-organic framework was proposed by the zeta potential and videos from an optical microscope.

## 2 Experimental sections

### 2.1 Materials and characterization

All reagents were purchased from commercial companies and used without further purification, including tetrakis (acetonitrile) copper(I) tetrafluoroborate (98%, AR), AgNO_3_ (purity: 99.8%), CdCl_2_ (>99%, AR), ZnSO_4_ (99.5%, AR), CuSO_4_.5H_2_O (>98%, AR), Copper(II) acetate monohydrate (>99%, AR), CuCl_2_.2H_2_O (99%, AR), CuCl (97%, AR), 3,5-dimethyl-1,2,4-triazole (97%, AR), 1-Hydroxy-7-azabenzotriazole (HOAT) (99%, AR), 2,6-Pyridinedicarboxylic acid (99%, AR), p-Phthalic acid (PTA) (99%), benzene-1,3,5-tricarboxaldehyde (purity:97%), and methanol (99.5%, AR).

Powder X-ray diffraction (XRD) measurements were carried out on a Rigaku D/max 2000 single-crystal diffractometer using Cu Kα radiation (λ = 0.15405 nm). Energy dispersive spectroscopy (EDS) analysis was performed using a HITACHI S-4800 scanning electron microscope (SEM) equipped with an Oxford X-max spectrometer. Fourier-transform infrared spectroscopy (FT-IR) was performed on NEXUS 470. The morphology of the samples was analyzed by a HITACHI S-4800 SEM and a JEM-2010 transmission electron microscope (TEM). The surface compositions of the samples were measured by X-ray photoelectron spectroscopy (XPS, Versa Probe PHI 5000). The absorption spectra of the samples were recorded using a Varian Cary 500 UV-visible spectrophotometer. N_2_ adsorption-desorption isotherms were obtained on a NOVA 4000 at 77 K, from which the specific surface area (*S*_*BET*_), pore volume (*V*_*P*_), and average pore diameter (*D*_*P*_) were calculated by using the BJH method.

### 2.2 Synthesis of various Cu-MOFs nanoparticles

The Cu metal salt tetrakis (acetonitrile) copper(I) tetrafluoroborate (CuSO_4_.5H_2_O/Copper(II) acetate monohydrate/CuCl_2_.2H_2_O/CuCl, 0.2961 ± 0.0005 g) was added into *X* mL of methanol for dissolution. The appropriate amount of 3, 5-dimethyl-1,2,4-triazole ligand ((1-Hydroxy-7-azabenzotriazole) HOAT/2,6-Pyridinedicarboxylic acid/(Terephthalic acid) PTA/benzene-1,3,5-tricarboxaldehyde, n_metal salt_: n_ligands_ = 1:1) was added to *Y* mL of methanol for dissolution (x + y = 25 mL). After being dissolved completely, the ligand solution was added to the metal sources solution. Then, it was sealed with plastic wrap, immersed in water at 25°C, and stirred for 3 h. The plastic wrap was removed, and then immersed in water at 25°C, and stirred for 3 h again. It was further dried at 60°C for 12–24 h until the material was completely dry. The material was used for antimicrobial testing and characterization after being uniformly ground. The obtained samples using tetrakis (acetonitrile) copper(I) tetrafluoroborate, CuSO_4_.5H_2_O, Copper(II) acetate monohydrate, CuCl_2_.2H_2_O, and CuCl were denoted as Cu-MOF, Cu-1-MOF, Cu-2-MOF, Cu-3-MOF and Cu-4-MOF, respectively.

### 2.3 Synthesis of various metal-MOFs nanoparticles

The synthesized procedure was similar to the development of Cu-MOFs, except the metal salts of Cu were replaced by AgNO_3_, CdCl_2_, and ZnSO_4_, respectively. The organic ligand was 3, 5-dimethyl-1,2,4-triazole was used in each instance. The obtained samples synthesized using AgNO_3_, ZnSO_4_, and CdCl_2_ were denoted as Ag-MOF, Zn-MOF, and Cd-MOF, respectively.

### 2.4 Synthesis of Cu-MOFs with various ligands

The synthesized procedure was similar to the development of Cu-MOFs, except the organic ligands of 3, 5-dimethyl-1,2,4-triazole were replaced by HOAT, 2,6-pyridinedicarboxylic acid, PTA, and benzene-1,3,5-tricarboxaldehyde, respectively. The Cu metal salt used was tetrakis (acetonitrile) copper(I) tetrafluoroborate. The obtained samples synthesized by HOAT, 2,6-Pyridinedicarboxylic acid, PTA, and benzene-1,3,5-tricarboxaldehyde were denoted as Cu-MOF-1, Cu-MOF-2, Cu-MOF-3, and Cu-MOF-4, respectively.

### 2.5 Culture and treatment of microorganisms

**Liquid medium:** 2.5 g tryptone, 5.0 g agar powder, 1.3 g yeast extract, and 2.5 g sodium chloride were added into 250 mL water in an Erlenmeyer flask and stirred for 10 min. Next, the pH value of this system was adjusted to between 7.2 and 7.4 with 1 mol/L sodium hydroxide solution. Finally, the mixture was disinfected in the high-handed sterilization pan to obtain a sterile and liquid medium.

**Solid medium:** 5.9 g agar powder was added into a 250 mL Erlenmeyer flask, and dissolved in 250 mL water by heating to 98°C. Next, the solution was sealed with a rubber plug and cooled to room temperature. Finally, the system was disinfected in the high-handed sterilization pan to obtain a sterile and solid medium.

**Culture of microorganisms:** The antibacterial activity of the samples was investigated using the agar well diffusion method. The Gram-positive *S. aureus* (ATCC6835), Gram-negative *E. coli* (ATCC8099), and *A. baumannii* (ATCC19606) were used in this work. The zone of inhibition test was employed to evaluate the antibacterial activity of the various Cu-MOF catalysts. First, Gram-positive *S. aureus*, Gram-negative *A. baumannii,* and *E. coli* were cultivated in the Luria-Bertani (LB) media and cultured at 37°C for 24 h. For the agar well diffusion method, initially, plates containing agar were prepared. Then, the nutrient agar plates were inoculated with 1 mL of bacterial suspension containing approximately 10^4^ colony forming units (CFU) using the spread plate method, and wells with 8 mm diameters were created on the plates using a sterilized stainless steel cork borer. Next, each of the MOF nanocatalysts (0.050 g) was inoculated into the wells and placed under visible light and dark conditions for 10 min, respectively. After that, these dishes were cultivated in the artificial bioclimatic test chamber for 24 h. Finally, these tests were performed twice and pure metal sources and ligands were used as control.

### 2.6 The videos from an optical microscope for anchoring bacteria

The images and videos from an optical microscope were used to examine the bacterial anchoring ability of Cu-MOF. Approximately 3 mg catalyst, assisted by ultrasound, was uniformly dispersed in 1.0 mL of bacterial suspension (1.5 × 10^7^ CFU of *E. coli*, OD = 0.1), then a drop of the above solution was placed under a microscope to obtain the videos under dark conditions.

## 3 Results and analysis

### 3.1 Characterization of samples

**FIGURE 1:**
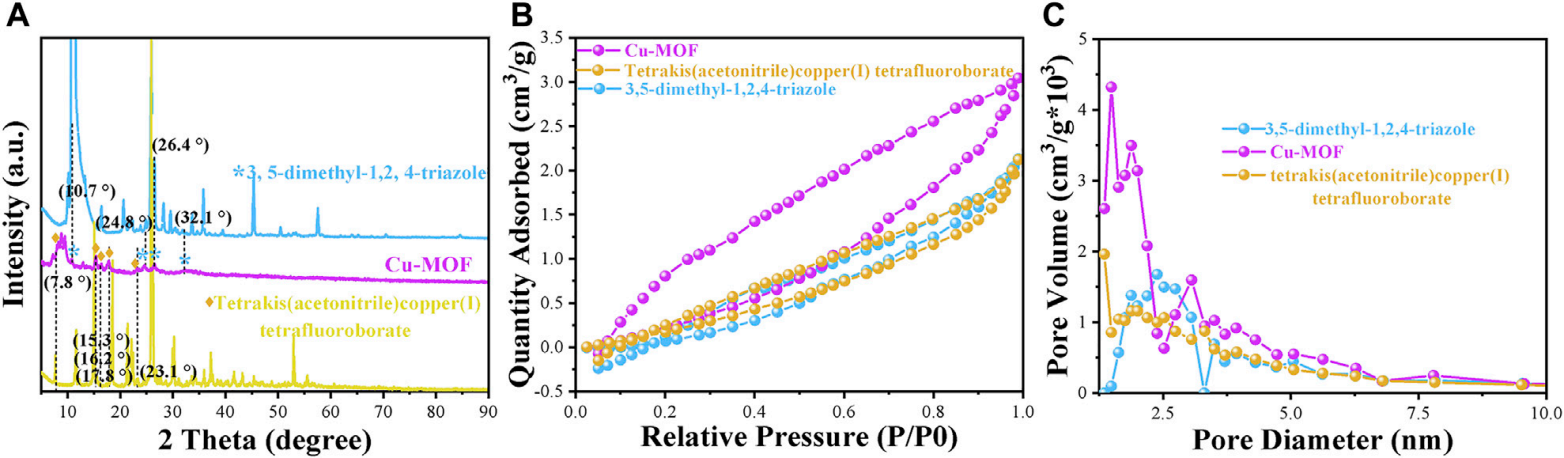
**(A)** XRD patterns, **(B)** N_2_ adsorption-desorption isotherms, and **(C)** pore size distribution of different powder samples of Cu-MOF, 3,5-dimethyl-1,2,4-triazole and tetrakis (acetonitrile) copper (I) tetrafluoroborate powder, respectively.

**FIGURE 2:**
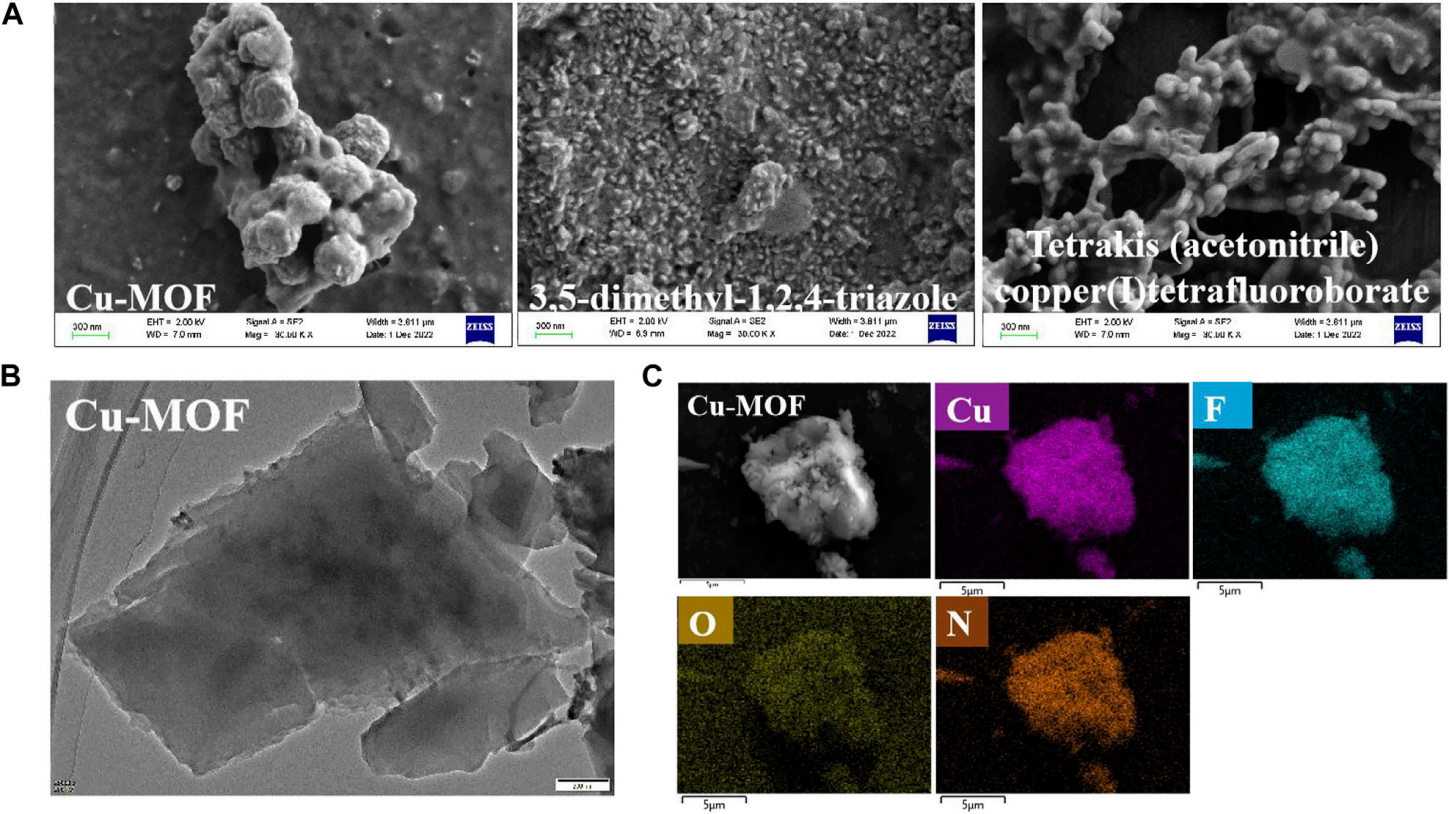
**(A)** SEM images of different powder samples of Cu-MOFs, 3,5-dimethyl-1,2,4-triazole and tetrakis (acetonitrile) copper (I) tetrafluoroborate powder, respectively, **(B)** TEM and **(C)** SEM images of Cu-MOFs with element mapping images.

The X-ray diffraction (XRD) patterns of Cu-MOF, 3, 5-dimethyl-1, 2, 4-triazole, and tetrakis (acetonitrile) copper(I) tetrafluoroborate powder, shown in Figure 1A, were utilized to validate the phase purity and crystalline structure of the nanomaterials. The characteristic peaks of Cu-MOF at 10.7°, 24.8°, 26.4°, and 32.1° were ascribed to the characteristic peaks of 3,5-dimethyl-1,2,4-triazole. The intense peaks at 7.8°, 15.3°, 16.2°, 17.8°, and 23.1° were assigned to tetrakis (acetonitrile) copper(I) tetrafluoroborate. This indicated that Cu-MOF was synthesized successfully and the crystal structure of Cu-MOF contains cuprous units bridged by 3, 5-dimethyl-1, 2, 4-triazole ligands. Furthermore, SEM images, shown in Figure 2A, revealed the morphology of Cu-MOF, 3, 5-dimethyl-1, 2, 4-triazole, and tetrakis (acetonitrile) copper(I) tetrafluoroborate powder, respectively. These images evidenced that the morphology of tetrakis (acetonitrile) copper(I) tetrafluoroboratethe was nano-tree-like, while 3,5-dimethyl-1,2,4-triazole powder was not. In addition, Cu-MOF exhibited a nanospheres morphology with plenty of obvious pores. The TEM images in Figure 2B clearly show that Cu-MOF presented a nanosheet morphology and was distributed in layers. The N_2_ adsorption-desorption isotherms of Cu-MOF, 3, 5-dimethyl-1, 2, 4-triazole and tetrakis (acetonitrile) copper(I) tetrafluoroborate powder are presented in Figure 1B and their corresponding structure parameters are collected in Supplementary Table S1. It is notable noticed that the isotherms of the above materials and the pore size distribution parameters indicate a microporous structure in Cu-MOF. The pore size distribution parameters are shown in Figure 1C and Table 1. The pore diameter of Cu-MOF was approximately 1.488 nm, belonging to the micropores (Kim et al., 2016). Different from Cu-MOF, the 3, 5-dimethyl-1, 2, 4-triazole and tetrakis (acetonitrile) copper(I) tetrafluoroborate, appeared to have larger pores of 2.384 nm and smaller diameter of 1.349 nm. In addition, Cu-MOF also exhibited the largest specific surface area (11.064 m^2^/g) and pore volume (0.008 cm^2^/g), compared with 3, 5-dimethyl-1, 2, 4-triazole and tetrakis (acetonitrile) copper(I) tetrafluoroborate. This was mainly ascribed to the fact that their pores were induced by the stacking of materials, while the microporous structure of Cu-MOF was formed during the growth of the crystal.

**FIGURE 3:**
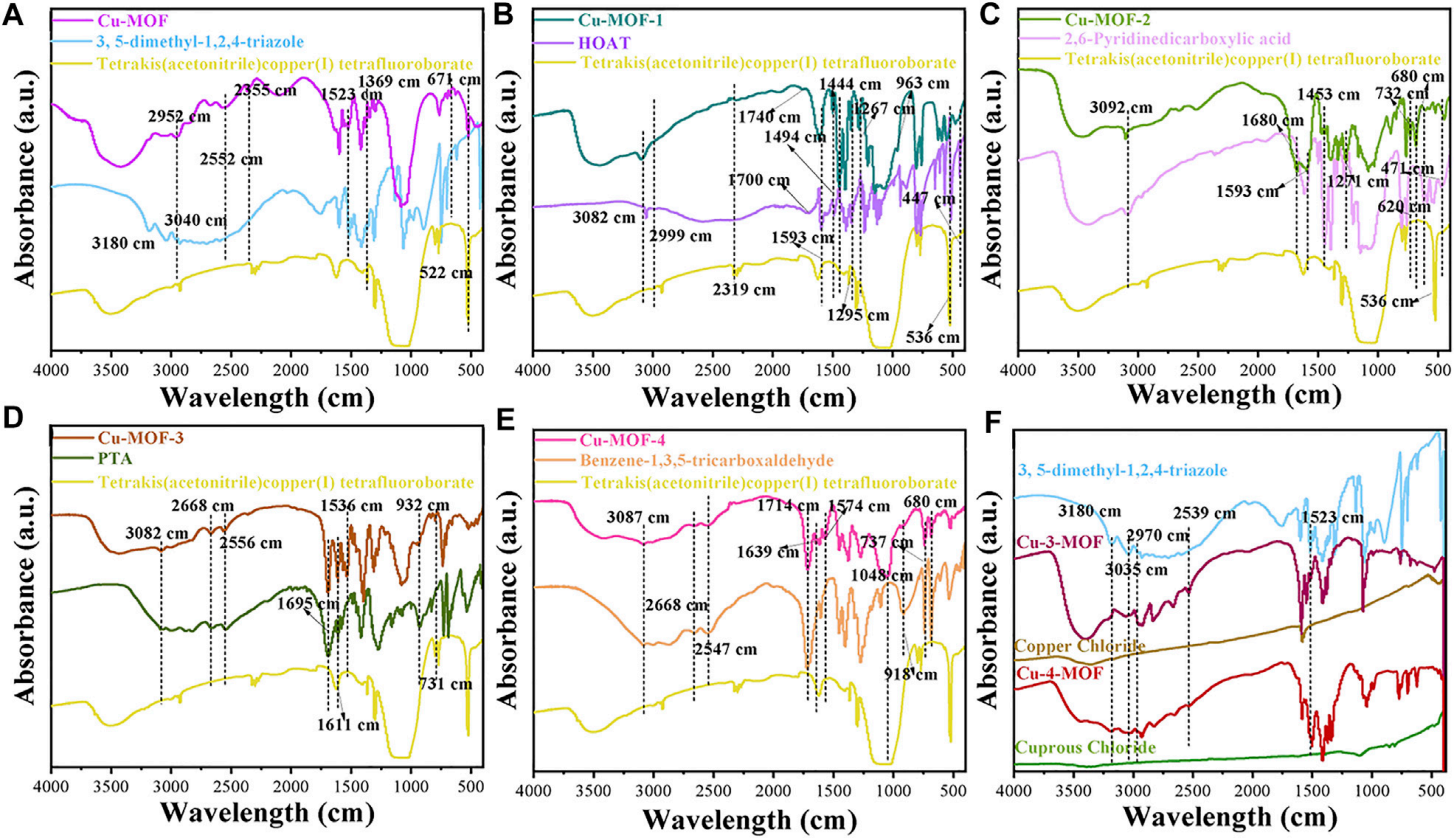
FTIR spectra of **(A)** Cu-MOF, **(B)** Cu-MOF-1, **(C)** Cu-MOF-2, **(D)** Cu-MOF-3, and **(E)** Cu-MOF-4 with their organic ligands of 3, 5-dimethyl-1, 2, 4-triazole, HOAT, 2,6-pyridinedicarboxylic acid, PTA and benzene-1, 3, 5-tricarboxaldehyde, and metal source of tetrakis (acetonitrile) copper (I) tetrafluoroborate, respectively, and **(F)** Cu-3-MOF and Cu-4-MOF.

**FIGURE 4:**
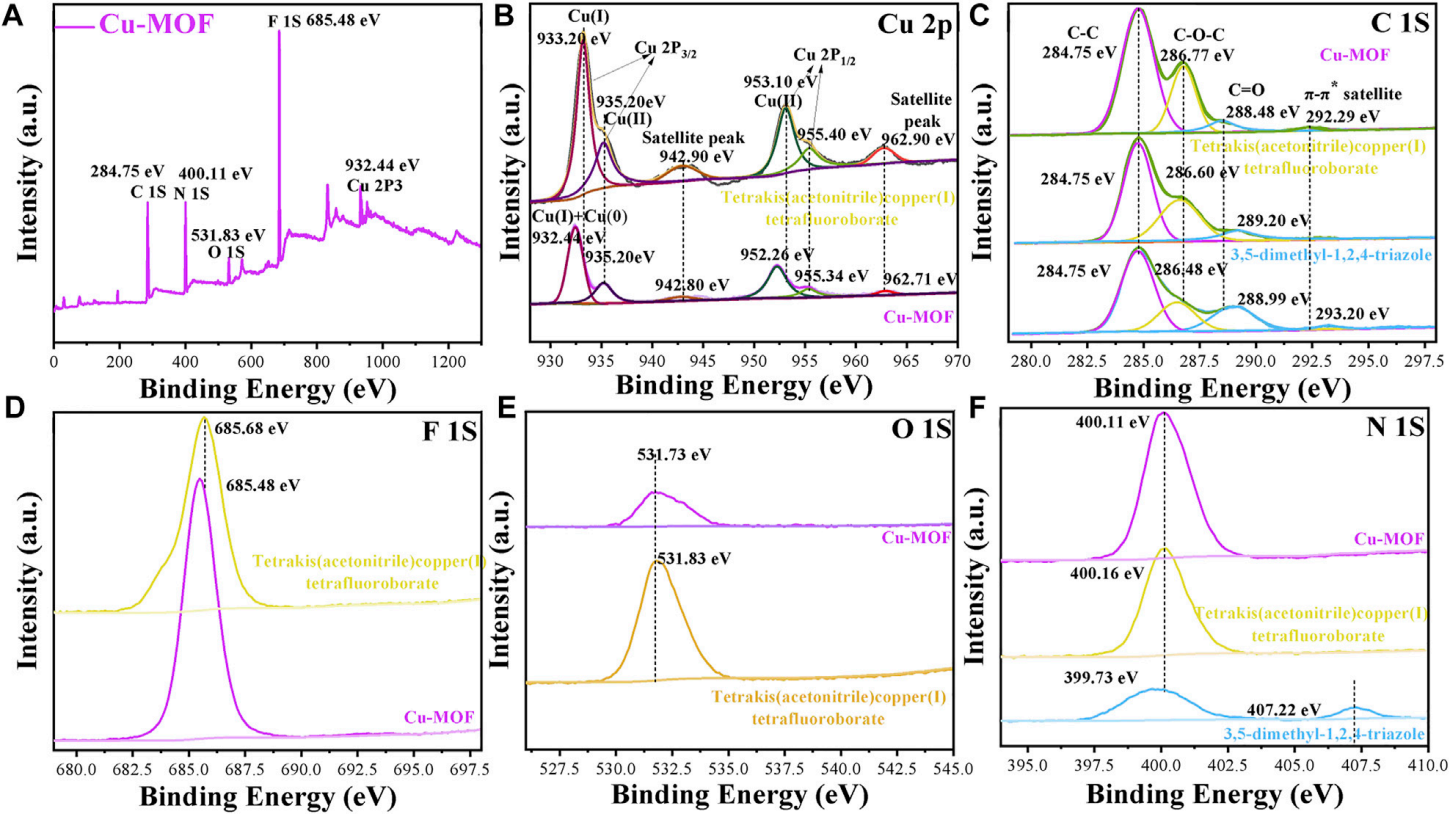
XPS spectra of **(A)** survey **(B)** Cu 2P, **(C)** C 1S, **(D)** F 1S and **(E)** O 1S and **(F)** N 1S in Cu-MOFs, 3, 5-dimethyl-1, 2, 4-triazole and tetrakis (acetonitrile) copper (I) tetrafluoroborate powder, respectively.

To initially evaluate the elemental composition and distribution, EDS analysis was conducted, as shown in Figure 2C. It demonstrated that Cu-MOF contained Cu, F, O, and N without other elements. It was observed that the Cu and N elements were distributed uniformly in the EDS mapping, implying that Cu bonded with 3, 5-dimethyl-1, 2, 4-triazole ligands in the Cu-MOF. FTIR spectra, shown in Figure 3, was performed to further test the binding mode and chemical bond between copper and an organic ligand. Compared with pure 3, 5-dimethyl-1, 2, 4-triazole and tetrakis (acetonitrile) copper(I) tetrafluoroborate, Figure 3A demonstrates that the bands at 2355, 1369, 1057, and 522 cm^-1^ of Cu-MOF were ascribed to the characteristic peaks of tetrakis (acetonitrile) copper(I) tetrafluoroborate and the peaks at 1523 and 671 cm^-1^ could be attributed to the deformation vibration and out-of-plane deformation vibration of N-H in 3, 5-dimethyl-1, 2, 4-triazole (Beenken et al., 2014). Additionally, the 3180 and 3040 cm^−1^ of tertiary amine bonds in 3, 5-dimethyl-1, 2, 4-triazole could be clearly observed (Parıldar and Ibik, 2013), while there were no obvious peaks in Cu-MOF. Furthermore, new bonds from 2552 to 2952 cm^−1^ appeared and were ascribed to tertiary amine bonds (Parıldar and Ibik, 2013). The intensity of the characteristic peaks at 1523 and 671 cm^−1^ derived from deformation vibration and out-of-plane deformation vibration of N-H, which was weaker in Cu-MOF. From this, we conclude that the Cu in tetrakis (acetonitrile) copper(I) tetrafluoroborate integrates with all the N atoms in 3, 5-dimethyl-1, 2, 4-triazole, while the Cu is mainly coordinated with tertiary amines of the above ligand (structure can be found in Supplementary Scheme S1). The spectrum of Cu-3-MOF and Cu-4-MOF, shown in Figure 4F, were analyzed to investigate the different coordination capabilities of cuprous and cupric sources with the same organic ligand. It was observed that the intensity of both the tertiary amine bonds from 2539 to 2970 cm^−1^ and the characteristic peaks of out-of-plane deformation vibration of N-H (1523 cm^−1^) became stronger in Cu-3-MOF than Cu-4-MOF, compared with the pure ligand. However, few characteristic tertiary amine bonds located at 3180 and 3035 cm^−1^ could be observed. It proved that cupric salt was more likely to form tertiary amine bonds with N-H than cuprous salt and both these salts were not able to unite with all the N in 3,5-dimethyl-1,2,4-triazole. It was proposed that the different coordinate atoms induced various coordination abilities with metals and it was more beneficial for N to coordinate with metal than O (Brodowska et al., 2016). To further investigate the chemical bond and coordination ability between the various organic ligands and Cu in tetrakis (acetonitrile) copper(I) tetrafluoroborate, the HOAT, 2,6-pyridinedicarboxylic acid, PTA, and benzene-1, 3, 5-tricarboxaldehyde were introduced. As can be seen in Supplementary Scheme S1, although the HOAT presented a pyridine structure with the same three N atoms compared with 3,5-dimethyl-1,2,4-triazole, the steric hindrance effect should be considered when N coordinated with Cu. Different from HOAT, there were two carboxyl groups and one N atom in the 2,6-pyridinedicarboxylic acid structure. The N atom was gradually replaced by carboxyl groups, presented in the structure of PTA and benzene-1, 3, 5-tricarboxaldehyde, respectively.

It was clearly observed that peaks around 2999, 2319, 1444, 1295, and 536 cm^−1^ were derived from the characteristic peaks of tetrakis (acetonitrile) copper(I) tetrafluoroborate. The bonds at 1593, 1494, 1444, 3082, and 447 cm^−1^ corresponded with the characteristic absorption peaks of HOAT in Cu-MOF-1. Furthermore, the peaks at 1593, 1494, and 1444 cm^−1^ derived from the pyridine ring of HOAT (Al-Saif et al., 2020), which could prove that there was no chemical bond between Cu and N in the pyridine ring. This also demonstrated that the peaks at 1267 and 963 cm^−1^ of Cu-MOF-1 should be ascribed to the telescopic vibration peak of N-O-H (Nikolić et al., 2008) and N-O (Alt et al., 2006), respectively, indicating a coordinate bond does not exist between the Cu and -N-OH. However, the characteristic peaks of 1740 cm^−1^ of Cu-MOF-1 for the -CH = N^+^-H- bond appeared when bonded with HOAT whose bond was around 1700 cm^−1^, which could conclude that the structure of -CH = N^+^-OH- exists when Cu coordinated with HOAT. However, no obvious peaks at 2240–2260 cm^−1^ (-N=N-OH) (Lijun Wu, 2013) could be observed. This demonstrates that Cu was more reluctant to coordinate with the N atom in position 8 of HOAT. The proposed coordination route of Cu with HOAT is shown in Supplementary Scheme S1. It was possibly ascribed to a large steric hindrance that hinders the coordination of copper and nitrogen of pyridine ring and position 7 of HOAT.

To explore whether the coordination ability of N with Cu was stronger than O, FTIR spectra of Cu-MOF-2 were performed, as shown in Figure 3C. The peaks at 3092, 1453, 6200, and 471 cm^−1^ well matched the characteristic absorption peaks of 2, 6-Pyridinedicarboxylic acid and the 536 cm^−1^ derived from Cu source in Cu-MOF-2, respectively. Meanwhile, the peaks at 3092 and 1271 cm^−1^ were ascribed to the carboxyl group of Cu-MOF-2 (Zhang X. et al., 2022), and the bond at 1593 cm^−1^ was attributed to the carboxylates formed by combing the Cu in tetrakis (acetonitrile) copper(I) tetrafluoroborate with the carboxyl group of 2,6-pyridinedicarboxylic acid (Papageorgiou et al., 2010), which confirms that only one carboxyl group in 2, 6-Pyridinedicarboxylic acid coordinates with Cu. Furthermore, the chemical bonds at 2510 and 1680 cm^−1^ were derived from the unsaturated amine (the structure can be referred to in Supplementary Scheme S1) formed due to the nitrogen in the pyridine ring coordinating with copper. The aforementioned could indicate that N is more propitious than O in forming a chemical bond with Cu, and there was only one carboxyl group used to cooperate with Cu and form copper carboxylate. Thus, for Cu-MOF-2, the O atom had a certain coordination ability with Cu. FTIR spectra of Cu-MOF-3 and Cu-MOF-4 synthesized by PTA and benzene-1, 3, 5-tricarboxaldehyde, indicating that more O atoms formed more Cu-O chemical bonds. It was clearly observed that the peaks at 3082, 2668, 2556, and 932 cm^−1^ matched well with the characteristic vibration peaks of the carboxyl group and -OH in PTA (Mohamadi et al., 2020). The peaks at 1695, 1611, and 731 cm^−1^ belonged to the 1, 4-disubstitution peak of Ar-COOH (Li et al., 2023). The peaks at 1536 cm^−1^ were derived from the copper carboxylate of Cu-MOF-3. Additionally, the chemical bonds at 3087, 2668, 2547, and 918 cm^−1^ corresponded with the vibration peaks of the carboxyl group and -OH in benzene-1, 3, 5-tricarboxaldehyde. The peaks at 1714, 737, and 680 cm^−1^ were ascribed to the 1, 3, 5-trisubstituted peaks of Ar-COOH (AkbarBandari et al., 2021). The peaks at 1574 cm^−1^ and 1639 cm^−1^ were derived from the copper carboxylate of Cu-MOF-4. Both Cu-MOF-3 and Cu-MOF-4 exhibited the copper carboxylate and carboxyl group at the same time, revealing that copper limited the coordination capability with oxygen even if benzene-1, 3, 5-tricarboxaldehyde had more O atoms than PTA. Meanwhile, the intensity of the peaks for copper carboxylate were stronger in Cu-MOF-3 than that in Cu-MOF-4. This was mainly ascribed to the fact that steric hinderance effect of the carboxyl group in benzene-1, 3, 5-tricarboxaldehyde was more prominent than in PTA. The results show that the N atoms were more prominent in forming chemical bonds with Cu than O atoms. More N atoms in the organic ligand could induce higher Cu content in the Cu-MOF without obvious steric hinderance effect, beneficial for the sterilization of bacteria after contact with the catalysts.

XPS spectra were carried out, as shown in Figure 4, to further investigate the micro components of Cu-MOF and observe the valence of different elements. Figure 4A shows the survey spectra of Cu-MOF. It demonstrates that Cu, N, O, and F coexisted in the sample, consistent with the EDS in Figure 2B and further indicating the successful synthesis of the Cu-MOF composites. The valence of the Cu species is clearly observed from the fitting curves of Cu-MOF and tetrakis (acetonitrile) copper(I) tetrafluoroborate. As can be seen in Figure 4B, the positions of Cu(Ⅰ) (932.5 eV) and Cu(0) (932.3 eV) of Cu 2p_3/2_ were too close to distinguish from each other in Cu-MOF (Ding et al., 2020). In addition, the characteristic Cu 2p_3/2_ and Cu 2p_1/2_ peaks of Cu(Ⅱ) for Cu-MOF were located at 935.20 eV, with its satellite peak of 942.80, and 952.26 eV (Zhang et al., 2017) with its satellite peak of 962.71 eV, respectively (Bulushev et al., 2017). Compared with Cu-MOF, all characteristic peaks of pure tetrakis (acetonitrile) copper(I) tetrafluoroborate exhibited a positive shift. This implied that the binding force between tetrakis (acetonitrile) copper(I) tetrafluoroborate and Cu-MOF existed, and the electrons were transferred from the Cu source to the Cu-MOFs (Hou et al., 2016). Meanwhile, all the characteristic F, O, and N peaks of tetrakis (acetonitrile) copper(I) tetrafluoroborate were at a higher binding energy than Cu-MOF, which could be seen in Figures 4D, E. However, the characteristic N peak of 3, 5-dimethyl-1, 2, 4-triazole (399.73 eV) was inferior to both tetrakis (acetonitrile) copper(I) tetrafluoroborate (400.16 eV) and Cu-MOF (400.11 eV), indicating the transfer of electrons from the Cu source to the organic ligand in Cu-MOF. The C 1s XPS spectra of the Cu-MOF, tetrakis (acetonitrile) copper(I) tetrafluoroborate, and 3, 5-dimethyl-1, 2, 4-triazole are shown in Figure 4C. The first peak at 284.75 eV was consistent with C atoms in the Cu-MOF, and the second bond located at 286.77 eV reflected the C-O-C chemical bond, induced by binding oxygen with a single C atom in Cu-MOF. The C1S peak at 288.48 eV represented the carbon atoms doubly bonded to oxygen atoms (Veyan et al., 2018) and the last bond around at 292.29 eV showed a π-excitation inherently peculiar to sp^2^-bonds of carbon (D'Acunto et al., 2018). It was evidenced that the bond for C-O-C (286.77 eV) of Cu-MOF was superior to that of pure tetrakis (acetonitrile) copper(I) tetrafluoroborate and 3, 5-dimethyl-1, 2, 4-triazole, respectively. This was mainly due to the C atoms bonded with O atoms being more electronegative. More electrons transferred from the C to the O atoms and induced a higher binding energy, due to more C-O-C chemical bonds existing in Cu-MOF. In contrast, the peaks of C=O in Cu-MOF (288.48 eV) were inferior to pure 3, 5-dimethyl-1, 2, 4-triazole (288.99 eV) but outperformed tetrakis (acetonitrile) copper(I) tetrafluoroborate. This was ascribed to the fact that C=O tends to decrease when Cu-MOF is synthesized. Similar to the tendency of C = O, the bonds of π-excitation of Cu-MOF (292.29 eV) presented a lower binding energy compared with 3, 5-dimethyl-1, 2, 4-triazole (293.20 eV). This was mainly due to the less π-excitation bonds appearing when Cu coordinated with 3,5-dimethyl-1,2,4-triazole. This could be explained by the FTIR spectra in Figure 3 and the possible structure of Cu-MOF can be observed in Supplementary Scheme S1.

XPS spectra of Cu-3-MOF and Cu-4-MOF were performed to analyze whether the valence state of the Cu source influenced the coordination ability with 3, 5-dimethyl-1, 2, 4-triazole. From the survey spectra in Supplementary Figures S1A, B, it can be concluded that Cu, N, O, and F coexist in both Cu-3-MOF and Cu-4-MOF samples, which is well matched with the structure of Cu-MOFs. As shown in Supplementary Figure S1C, the peak of 932.4 eV for Cu-4-MOF was derived from the prominent characteristics of Cu(Ⅰ) and Cu(0) of the Cu 2p_3/2_ peak. The Cu(Ⅱ) and its satellite peak were located at 933.44 and 942.80 eV, respectively. The peaks at 952.06 and 954.60 eV belonged to the Cu(Ⅱ) of the Cu 2p_1/2_ peak and their satellite peak was at 962.50 eV. Compared with Cu-4-MOF, all the characteristic peaks of N, O, and F in Cu-3-MOF presented a higher binding energy. The difference in N peaks was especially obvious between the Cu-4-MOF (399.20 eV) and Cu-3-MOF (400.28 eV). This was ascribed to the fact that the copper ions in Cu-3-MOF coordinated with more N elements than the cuprous ions in Cu-4-MOF and induced more electron transfer from Cu to N, resulting in the higher binding energy of Cu-3-MOF. This could be further explained by the FTIR in Figure 3 and the analysis of C1S peaks of Cu-3-MOF and Cu-4-MOF in Supplementary Figure S1G. From the C1S spectra, it can be found that both these two MOFs exhibited the characteristic peaks of C-C, C-O-C, and C = O, and their binding energy of Cu-3-MOF was superior to those of Cu-4-MOF. Besides this, there existed a peak consistent with the π-excitation in Cu-4-MOF, while no obvious peak was observed in Cu-3-MOF. This was attributed to the copper ions in Cu-3-MOF coordinating with more N elements than the cuprous ions in Cu-4-MOF, and inducing the conjugate structures of 3, 5-dimethyl-1, 2, 4-triazole in Cu-3-MOF, which was destroyed heavily. In conclusion, 3, 5-dimethyl-1, 2, 4-triazole was the most suitable organic ligand to form coordinate bonds with tetrakis (acetonitrile) copper(I) tetrafluoroborate than other ligands, namely, HOAT, 2,6-pyridinedicarboxylic acid, PTA and benzene-1,3,5-tricarboxaldehyde, due to there being more N atoms in 3, 5-dimethyl-1, 2, 4-triazole. Additionally, the copper ions provided a stronger coordination ability with N elements than the cuprous ions.

### 3.2 Antibacterial assay

**FIGURE 5:**
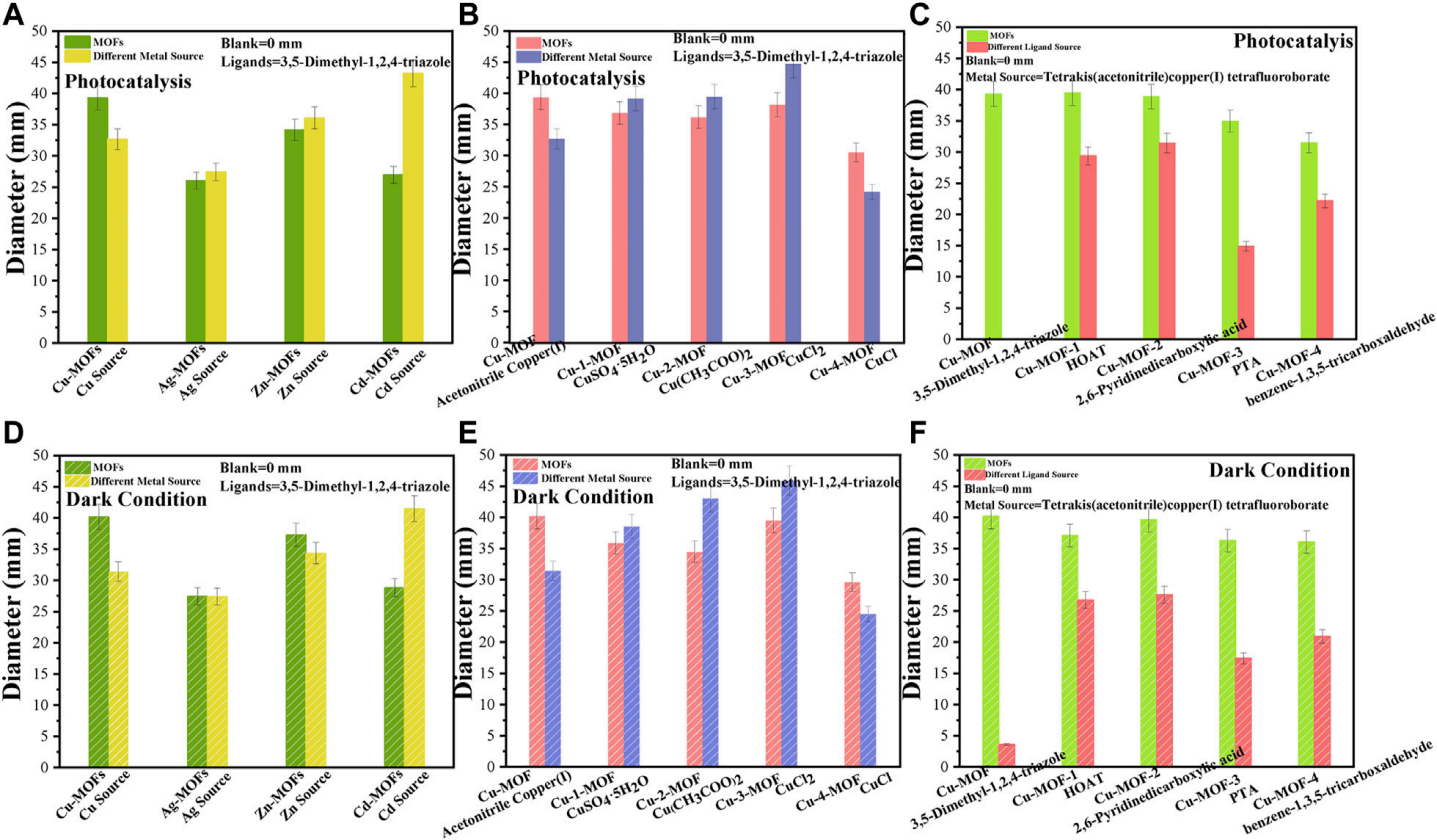
The zone of inhibition test of Cu-MOFs, Ag-MOFs, Zn-MOFs, and Cd-MOFs with their metal sources under **(A)** photocatalysis and **(B)** dark condition, of Cu-MOF, Cu-1-MOF, Cu-2-MOF, Cu-3-MOF and Cu-4-MOF with different metal sources under **(C)** photocatalysis and **(D)** dark conditions, and of Cu-MOF, Cu-MOF-1, Cu-MOF-2, Cu-MOF-3, and Cu-MOF-4 with different organic ligands under **(E)** photocatalysis and **(F)** dark conditions, in nutrient agar plates inoculated with a bacterial suspension of *S. aureus*.

**FIGURE 6:**
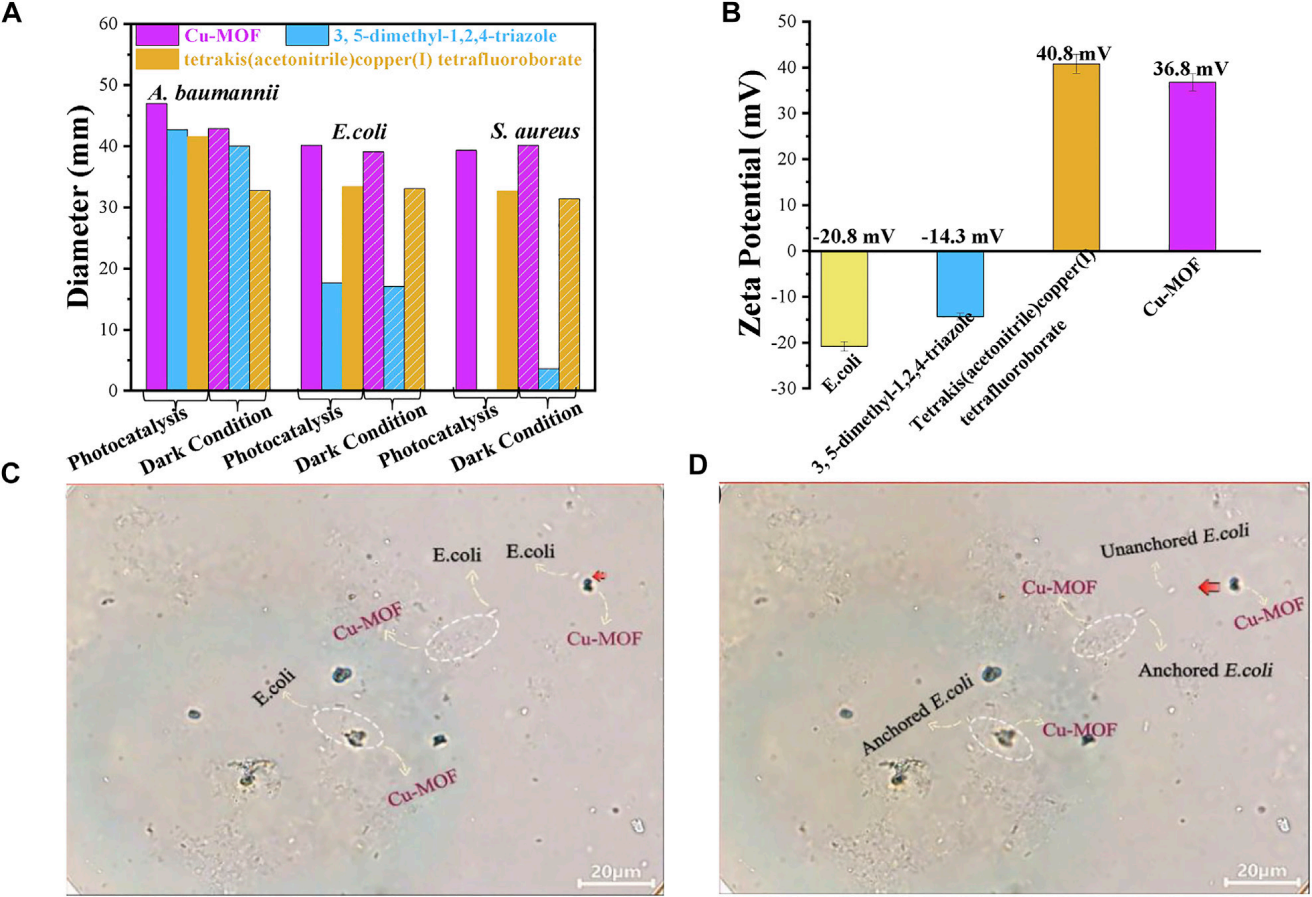
**(A)** The universal applicability testing of antimicrobials around Cu-MOFs for *S. aureus*, *A. baumannii,* and *E. coli* under photocatalysis and dark conditions, respectively. **(B)** Zeta potential of *E. coli* suspension and different samples. The screenshots from an optical microscope for **(C)** unanchored and **(D)** anchored *E. coli*.

The zone of inhibition test was executed against bacteria using metal-MOFs catalysts synthesized by various metal sources and organic ligands as the antibacterial agents. Figures 5A, B display the diameters of the inhibition zones formed around the Cu-MOF, Ag-MOF, Zn-MOF, and Cd-MOF discs under visible light irradiation and dark conditions, respectively, exploring the antimicrobial activity of some periodic metallic elements around copper. It was evidenced that the Cu-MOF generated an inhibition-zone diameter of 40.17 mm under dark conditions, significantly larger than that under visible light (39.32 mm). For the antibacterial effect of the Ag-MOF, it could be found that the inhibition-zone diameter under visible light was 26.05 mm, which showed little change when compared with that of the dark condition (27.44 mm). A weaker inhibition effect of Zn-MOF compared to Cu-MOF was shown, as can be seen in Figure 6. Zn-MOF displayed a diameter of 34.21 mm when irradiated by visible light, which was smaller than that under dark conditions (37.32 mm). Cd-MOF, synthesized by the Cd atom which is located in the second subfamily and below the Zn atom, demonstrated its antibacterial effect. There was little difference between its inhibition effect of photocatalysis (26.97 mm) and dark conditions (28.83 mm). The results exhibited that the inhibition activity of the MOFs developed using the metals of the first subfamily was superior to that of the second subgroup. For example, the inhibition zone diameter of Cu-MOF was larger than that of Zn-MOF, Ag-MOF, and Cd-MOF with similar activity. Meanwhile, it could also be observed that the antibacterial activity of the MOFs derived from metals of the same sub-family, the fourth circle was higher than that of the fifth circle. This is shown by the larger inhibition zone diameter of Cu-MOF (40.17 mm) and Zn-MOF (37.32 mm), while there were smaller diameters for Ag-MOF (27.44 mm) and Cd-MOF (28.83 mm), respectively. The reason may be ascribed to the fact that the ionic radius of Zn^2+^ (30 pm) is larger than that of Cu^2+^ (29 pm), while smaller than those of Ag^+^ (47 pm) and Cd^2+^ (48 pm) (Liu et al., 2020), respectively. The larger radius of metal ions was beneficial to coordinate with N elements (Gu and Lan, 2021), resulting in a more powerful inhibition effect of Cu-MOF than other metal-MOFs. To investigate the effect of the chemical state of copper on the structure of Cu-MOF and its antibacterial activity, the inhibition-zone test of Cu-MOFs synthesized using other organic coppers (copper(II) acetate monohydrate), and inorganic coppers (CuSO_4_.5H_2_O, CuCl_2_.2H_2_O and CuCl) were performed, shown in Figures 5C, D, respectively. It was clear that Cu-2-MOF (Cu(CH_3_COO)_2_) showed a inhibition zone diameter of 36.16 mm under visible light, which was 1.76 mm larger than the diameter in dark conditions (34.40 mm). For Cu-1-MOF (CuSO_4_.5H_2_O), the bacteriostatic circle was 36.83 mm in diameter under visible light irradiation, and the dark conditions showed an inhibition zone of 35.82 mm. Cu-3-MOF (CuCl_2_.2H_2_O) registered inhibition zone diameters of 38.15 mm and 39.46 mm under visible light and dark conditions, respectively. Different from the Cu-3-MOF, Cu-4-MOF (CuCl) produced a maximum average inhibition zone diameter of 30.49 mm when being irradiated by visible light, a little larger than under dark conditions (29.56 mm). This was ascribed to the fact that organic ligands provide a more rigid connection mode, larger flexible three-dimensional space, and stronger coordination ability, compared with inorganic ligands. This was beneficial for regulating and modifying the structure of the Cu-MOFs (Yin et al., 2015). Finally, this could lead to a larger ratio of Cu elements in Cu-MOF when it is derived from organic ligands, presenting higher antibacterial activity.

The above results reveal that a Cu-MOF derived from organic coppers of tetrakis (acetonitrile) copper(I) tetrafluoroborate demonstrates an outstanding antibacterial performance. The inhibition abilities of Cu-MOF-1, Cu-MOF-2, Cu-MOF-3, and Cu-MOF-4 were compared to further investigate the coordination ability of various organic ligands, i.e., HOAT, 2, 6-pyridinedicarboxylic acid, PTA, and benzene-1, 3, 5-tricarboxaldehyde with tetrakis (acetonitrile) copper(I) tetrafluoroborate. As shown in Figure 5E, the Cu-MOF-1 (HOAT) created an inhibition-zone diameter of 39.45 mm under visible light, a little more than under dark conditions (37.08 mm, as shown in Figure 5F). Cu-MOF-2 demonstrated an inhibition effect of 38.91 and 39.06 mm when the bacteria reacted with the catalyst under photocatalysis and dark conditions, respectively. Similar observations of the sterilization ability of Cu-MOF-3 (PTA) were made. An inhibition-zone diameter of 34.93 mm under visible light irradiation was discovered, showing a significantly reduced diameter compared to that in dark conditions (36.30 mm). Cu-MOF-4 (benzene-1, 3, 5-tricarboxaldehyde) displayed a bacteriostatic circle diameter of 31.49 mm, which was smaller than that in the dark conditions (36.04 mm). These results proved that the 3,5-dimethyl-1,2,4-triazole was the most appropriate ligand to coordinate with tetrakis (acetonitrile) copper(I) tetrafluoroborate, when compared to HOAT, 2,6-Pyridinedicarboxylic acid, PTA, and benzene-1,3,5-tricarboxaldehyde organic ligands. During this research process of antibacterial properties, it was found that F had nearly no sterilization activity. We used the FTIR, activity test, and the scheme of Cu-MOF synthesized by Cu coordinated with various ligands of 3,5-dimethyl-1,2,4-triazole, HOAT, 2,6-Pyridinedicarboxylic acid, PTA and benzene-1,3,5-tricarboxaldehyde ligands to illustrate the maximum amount of Cu^2+^ ions to effectively achieve the highest antibacterial activity of Cu-MOF.

To investigate the universal applicability of antimicrobials for Cu-MOF, the antibacterial ability against *A. baumannii* and *E. coli* was investigated under visible light irradiation and dark conditions, as shown in Figure 6A. The inhibition-zone diameter for *S. aureus* treated by Cu-MOF, 3, 5-dimethyl-1, 2, 4-triazole, and tetrakis (acetonitrile) copper(I) tetrafluoroborate powder by photocatalysis was 39.32 mm, 0 mm, and 32.66 mm, respectively. This was different from their inhibition-zone diameters under dark conditions of 40.17 mm, 3.6 mm, and 31.35 mm, respectively. We also collected the data of the inhibition zone of *E. coli and A. baumannii* by using the same dosage of Cu-MOF, 3, 5-dimethyl-1, 2, 4-triazole and tetrakis (acetonitrile) copper(I) tetrafluoroborate powder. As shown in Figure 6A, the diameter of sterilization for *E. coli* was shown to be 40.14 mm, 17.64 mm, and 33.14 mm under visible light, and 39.09 mm, 17.12 mm, and 33.06 mm under dark conditions, respectively. The inhibition-zone diameter for *A. baumannii* was found to be 46.98 mm, 42.73 mm, and 41.59 mm under visible light, and 42.86 mm, 40.05 mm, and 32.79 mm under dark conditions, respectively. Cu-MOF had an excellent antibacterial ability against *S. aureus*, *A. baumannii,* and *E. coli*, and reveals the universal applicability of the antimicrobial Cu-MOF. The limited difference of the antimicrobial diameters under light irradiation to the dark conditions reveals that Cu-MOF is also suitable for the utilization of sterilization without light irradiation.

The antibacterial mechanisms were proposed based on the consideration of enriching the structure of Cu-MOF, inducing the maximum amount of Cu^2+^ ions to coordinate with the ligands as soon as possible, and enhancing the anchoring capability of Cu-MOF through electrostatic or hydrophobic interactions. The sterilization ability was strengthened by increasing the probability of copper ions coming into contact with bacteria. Initially, the elements of Zn, Ag, and Cd from the same period and sub-family around Cu were introduced to synthesize Zn-MOF, Ag-MOF, and Cd-MOF. This illustrated that the atomic structure characteristics of Cu were more beneficial for coordinating with ligands. Furthermore, the organic cupric source would have the strongest coordination capacity with the 3, 5-dimethyl-1, 2, 4-triazole when compared with other copper salts and the organic ligands with pyridine and carboxylic acid. These resulted in more copper ions and ligands interacting to construct the bridging relationship in Cu-MOF. Then, it effectively led to the death of more bacteria by exposing them to an increased number of copper ions. This is evidenced by the FTIR analysis in Figure 3 and the inhibition-zone test in Figure 5.

In addition, another antibacterial mechanism was put forward based on physical contact between the MOFs and bacteria *via* electrostatic interaction (Miao et al., 2019) or hydrophobic interactions (Pang et al., 2017) to generate effective antibacterial capability by cracking the cell membrane and inactivating the intracellular substance (Vecitis et al., 2010). Generally, the Cu^2+^ ions in solution released from Cu-MOFs are gathered on the surface of microorganisms. This was induced by electrostatic force from contradictory charges between the bacteria and Cu^2+^ ions if the bacteria accidentally encounter Cu^2+^ ions. Then, the charge balance of bacteria was destroyed, leading to serious cell collapse and final bacteria death by bacteriolysis. Furthermore, Cu ions could pass through the cell membrane and then enter the bacteria, leading to the final death of the bacteria. This was ascribed to the fact that the Cu^2+^ ions changed the structure and function of the protein by interacting with some functional groups on the protease, such as the sulfhydryl group (ASH), the amino group (ANH_2_), and the hydroxyl group (AOH) (Miao et al., 2017). However, it was beneficial to shorten the distance between Cu^2+^ ions and bacteria and increase the probability of bacteria being in contact with Cu^2+^ ions anchoring the bacteria to the Cu-MOFs *via* electrostatic interaction or hydrophobic interactions, leading to higher sterilization than killing bacteria randomly.

The Zeta potential of *E. coli* and different materials were analyzed, as shown in Figure 6B, to prove that another anchoring mechanism was the physical contact between the Cu-MOF and bacteria *via* electrostatic interaction. It was evidenced that the surface of *E. coli* presented negative charges of −20.8 mV. Furthermore, 3, 5-dimethyl-1, 2, 4-triazole presented more negative charges (−14.3 mV) than tetrakis (acetonitrile) copper(I) tetrafluoroborate (40.8 mV) but was more positive than bacteria. Opposite to *E. coli*, the surface potential of Cu-MOF was 36.8 mV after the 3, 5-dimethyl-1, 2, 4-triazole coordinated with tetrakis (acetonitrile) copper(I) tetrafluoroborate. This revealed that there existed an electrostatic attraction induced by the contradictory potential between *E. coli* and Cu-MOF. As a result, the distance between Cu^2+^ ions and bacteria could be shortened and the contact time could be prolonged, beneficial for promoting antibacterial efficiency. This was further explained by the video and screenshots from an optical microscope, shown in Supplementary Video S1 and Figures 6C, D. It was observed that Cu-MOFs catalysts were dispersed evenly in the bacteria suspension and plenty of bacteria was moving around the Cu-MOFs. Some anchored bacteria did not freely move, since these bacteria were fixed by the Cu-MOFs. Other unanchored cells could swim out of control and even moved far away from Cu-MOFs. This showed that the Cu-MOFs possessed the ability to capture the bacteria through the potential electrostatic attraction, consistent with the zeta potential shown in Figure 6.

**SCHEME 1:**
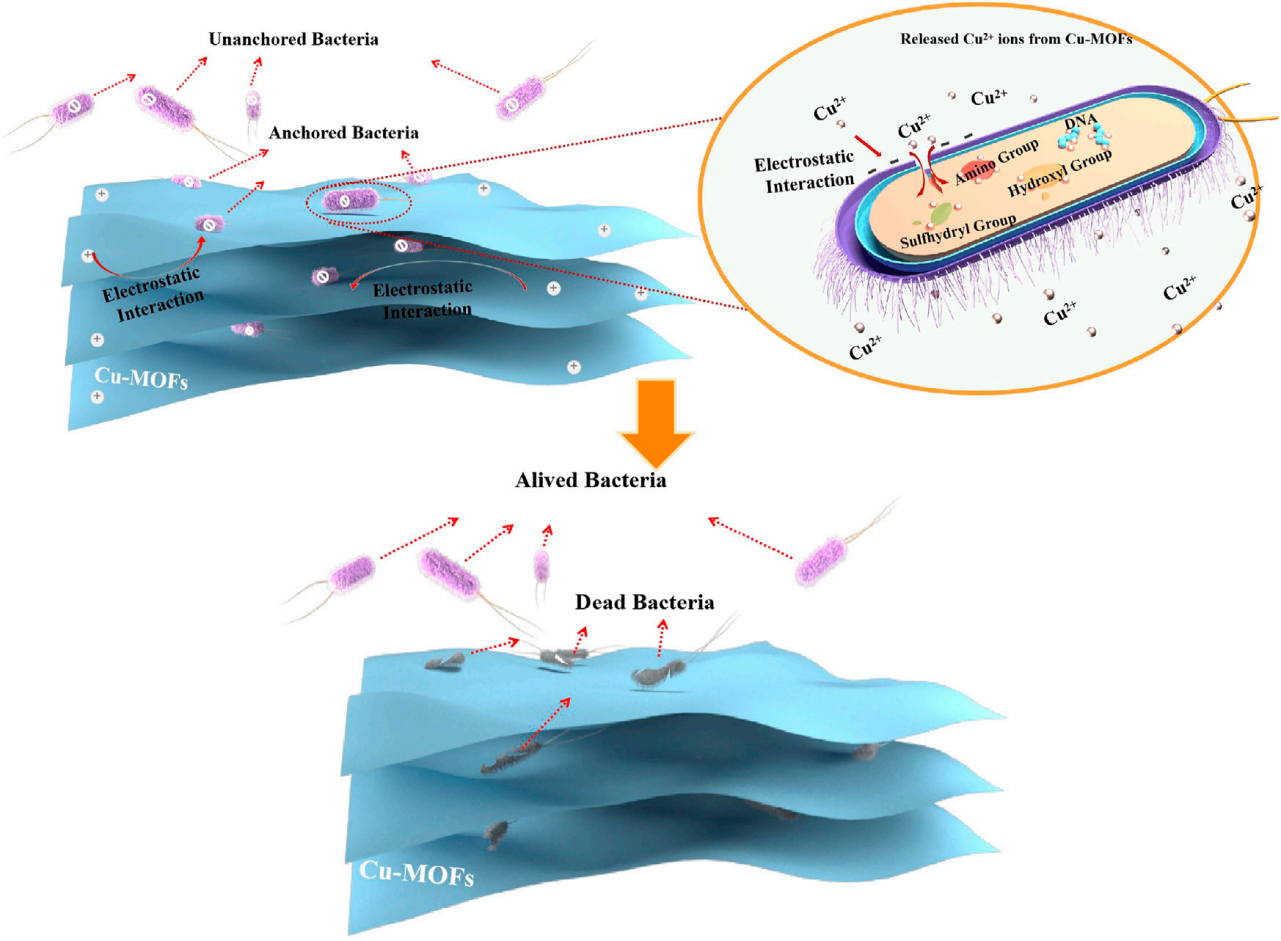
Illustration of antibacterial mechanism of Cu-MOFs.

In order to clearly elaborate on the antibacterial process, the antibacterial mechanism was proposed, which can be seen in Scheme 1. It is notable that the bacteria were anchored by Cu-MOFs *via* electrostatic interaction because of the opposite zeta potential between the bacteria and the material. Then the Cu^2+^ ions in solution released from Cu-MOFs could gather on the surface of the microorganisms. This was induced by electrostatic force from the contradictory charges between the bacteria and Cu^2+^ ions. Furthermore, Cu ions could pass through the cell membrane and then enter into the bacteria, leading to the final death of the bacteria. This was ascribed to the fact that the Cu^2+^ ions changed the structure and function of the protein by interacting with some functional groups on the protease, such as the sulfhydryl group (ASH), the amino group (ANH_2_), and the hydroxyl group (AOH). On the contradictory, the un-anchored bacteria by Cu-MOFs stayed alive if they did not touch the Cu^2+^ ions by accident.

## 4 Conclusion

In conclusion, the Cu-3, 5-dimethyl-1, 2, 4-triazole MOF (Cu-MOF) was successfully synthesized at room temperature and exhibited superior sterilization activity with the probe of bacteria of *S. aureus* during the dark system. XRD, SEM, TEM, BET, XPS, and FTIR were used to demonstrate the structural properties of the Cu-MOFs and the way Cu coordinates with organic ligands. These demonstrated that Cu-MOF was the most effective antibacterial agent among other transition metal elements of the IB and IIB sub-family such as Zn-MOF, Cd-MOF, and Ag-MOF. In addition, the organic cupric salt was more beneficial in regulating and modifying the structure of the Cu-MOF and induced a larger inhibition-zone diameter than inorganic cuprous-MOFs. Furthermore, Cu-MOF synthesized using imidazole ligand showed a better ability to kill bacteria, which was ascribed to the fact that there existed a strong coordinate capability between tetrakis (acetonitrile) copper(I) tetrafluoroborate and 3, 5-dimethyl-1, 2, 4-triazole compared to the other organic ligands of pyridine and carboxylic acid. The zeta potential and video of the process of anchoring bacteria proved that the bacteria was only anchored by Cu-MOFs *via* electrostatic interaction, leading to effective sterilization. Finally, the Cu-3, 5-dimethyl-1, 2, 4-triazole MOFs presented broad antimicrobial properties for *S. aureus*, *A. baumannii,* and *E. coli*, owing to their significant inactivation capability.

## Data Availability

The original contributions presented in the study are included in the article/Supplementary Material, further inquiries can be directed to the corresponding authors.
