# Supplementary material for: The synthesis and highly effective antibacterial properties of Cu-3, 5-dimethy l-1, 2, 4-triazole metal organic frameworks
Source: Front Chem. 2023 Feb 15;11:1124303. doi: 10.3389/fchem.2023.1124303 (PMC9974664; doi:10.3389/fchem.2023.1124303)
Supplement: Supplementary file 2 [file DataSheet1.docx]

Supplementary Material

**The Synthesis and Highly Effective Antibacterial Properties of Cu-3, 5-dimethyl-1,2,4-triazole Metal Organic Framework**

Xiaolin Xu^1#^, Mengna Ding^1#^, Kaiquan Liu^1^, Shiwen Yu^1^, Runxiang Du^1^, Yingchun Miao^1*^, Yanmi Liu^1^, Gong Ying^1^, Yuning Huo^2*^, Hexing Li^2*^

*** Correspondence:**

*Yingchun Miao. Phone, Fax: 86-21-6432-2272; email: yingchun1979@sohu.com.

*Yuning Huo. Phone, Fax: 86-21-6432-1673; email: [huoyuning@shnu.edu.cn](mailto:huoyuning@shnu.edu.cn)

**
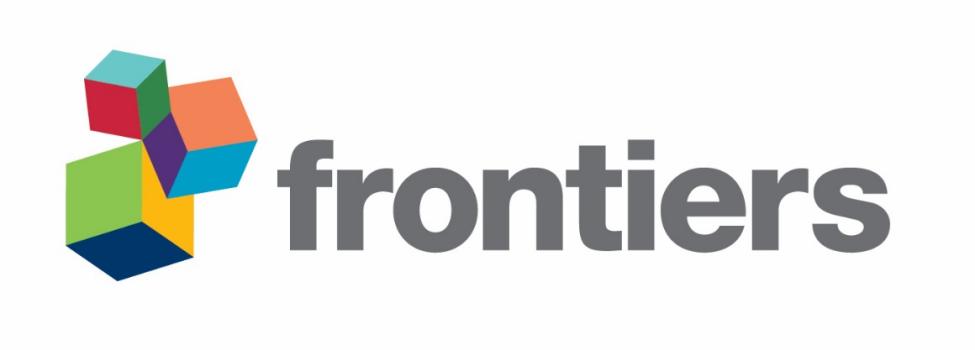
**

**Supplementary** Table 1. Structure parameters of Cu-MOF, 3,5-dimethyl-1,2,4-triazole and tetrakis(acetonitrile)copper(I) tetrafluoroborate powder, respectively.

| ***Sample*** | ***S_BET_* (m^2^/g)** | ***V_P_* (cm³/g)** | ***D_P_* (nm)** |
| --- | --- | --- | --- |
| Cu-MOF | 11.064 | 0.008 | 1.488 |
| 3,5-dimethyl-1,2,4-triazole | 5.198 | 0.005 | 2.384 |
| Tetrakis(acetonitrile)copper(I) tetrafluoroborate | 5.610 | 0.005 | 1.349 |


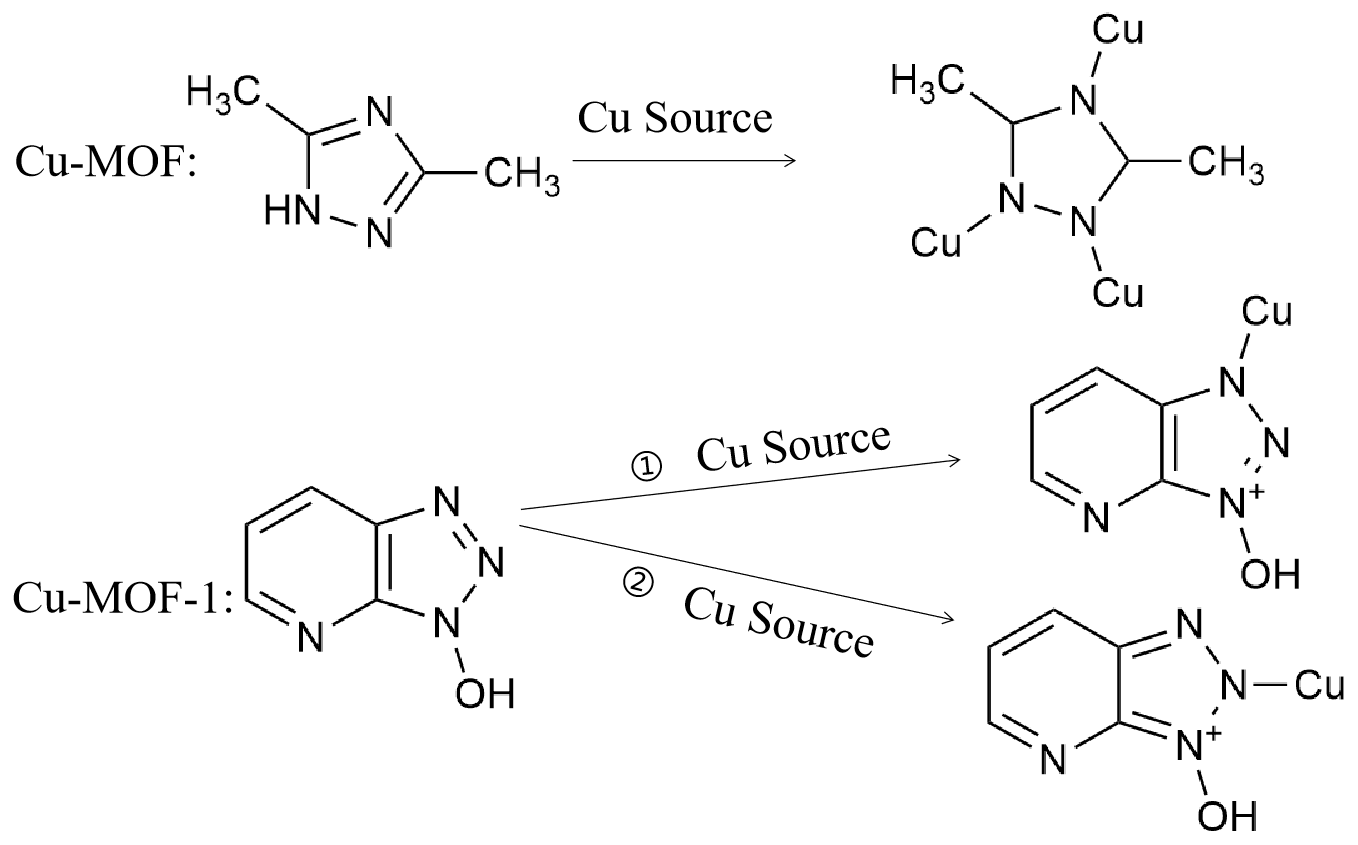

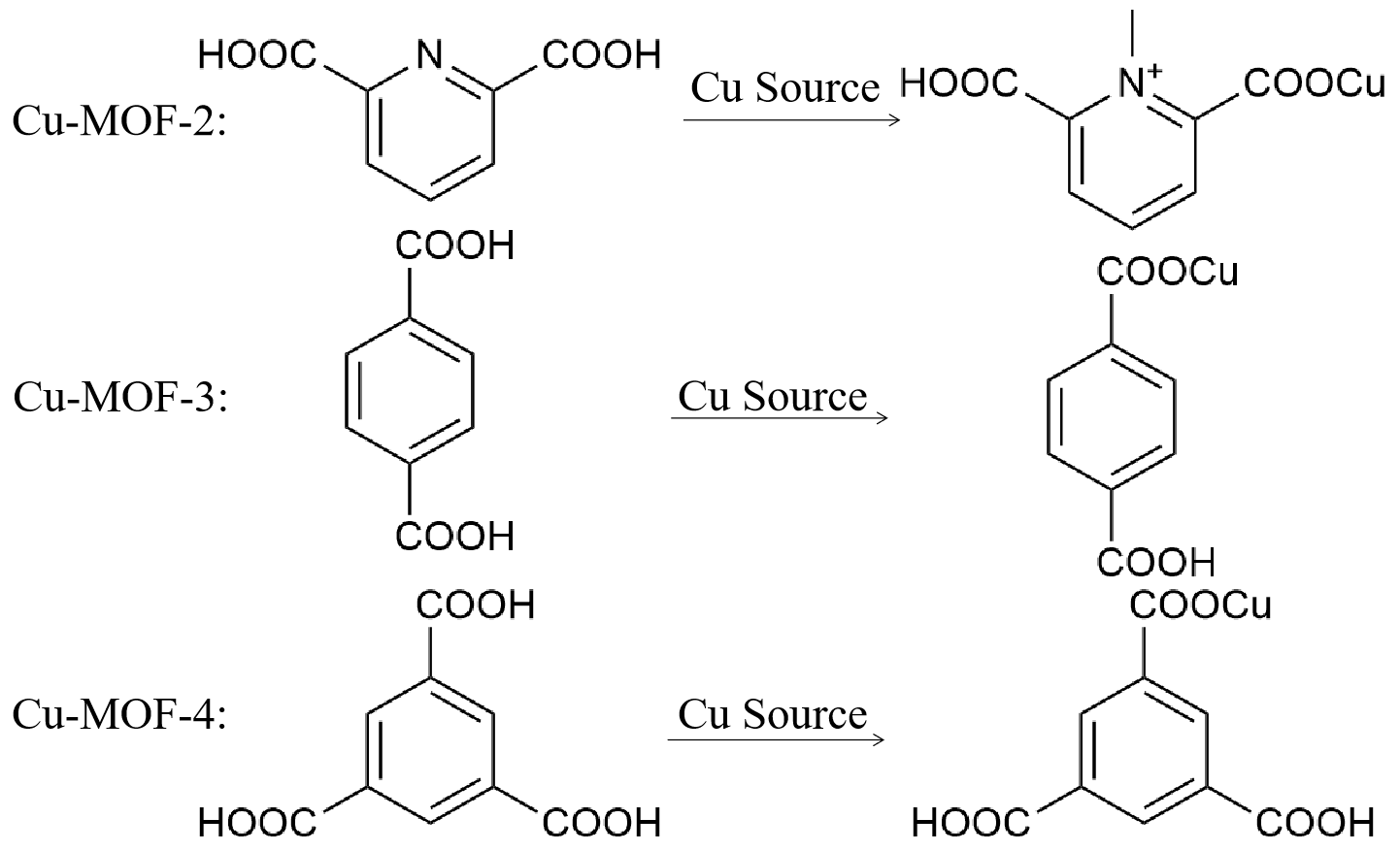


**Supplementary Scheme 1.** Illustration of Cu-MOF, Cu-MOF-1, Cu-MOF-2, Cu-MOF-3 and Cu-MOF-4 synthesized by Cu coordinated with various ligands of 3,5-dimethyl-1,2,4-triazole, HOAT, 2,6-Pyridinedicarboxylic acid, PTA and benzene-1,3,5-tricarboxaldehyde ligands, respectively.

**(C)**

**(B)**

**(A)**

**(F)**

**(E)**

**(D)**

**(G)**

**Supplementary Figure 1.** XPS of survey spectra of (A) Cu-3-MOF and (B) Cu-4-MOF, (C) Cu 2P, (D) F 1S, (E) O 1S, (F) N 1S and (G) C 1S in Cu-3-MOF and Cu-4-MOF powder, respectively.
